# Impostor Phenomenon and Its Relationship to Self-Esteem Among Students at an International Medical College in the Middle East: A Cross Sectional Study

**DOI:** 10.3389/fmed.2022.850434

**Published:** 2022-04-04

**Authors:** Maryam Jameel Naser, Nebras Ebrahim Hasan, Manal Hasan Zainaldeen, Ayesha Zaidi, Yusuf Mahdi Ahmed Mulla Hasan Mohamed, Salim Fredericks

**Affiliations:** School of Medicine, Royal College of Surgeons in Ireland, Medical University of Bahrain, Adliya, Bahrain

**Keywords:** imposter phenomenon, Clance's Imposter Phenomenon Scale, self-esteem, Rosenberg's Self-Esteem Scale, medical education

## Abstract

The atmosphere of constant scrutiny of academic ability that prevails in medical colleges may leave some students at risk of expressing feelings of intellectual fraudulence and phoniness. Impostor phenomenon (IP) traits have been associated with anxiety, depression, job dissatisfaction, and poor professional performance. Internationally trained junior doctors exhibit stronger IP feelings than colleagues trained within their own country of citizenship. These feelings may develop during student life. International universities are diverse and complex environments where students may be emersed in a cultural milieu alien to their societies of origin, leading to feelings of isolation. Individuals with IP traits often perceive themselves as the “only one” experiencing this phenomenon, resulting in further isolation and negative self-evaluation, especially among women and underrepresented minorities. IP has also been linked to low self-esteem among students. This study assessed the prevalence of IP and its relationship to self-esteem among students at a campus of a European medical college with a large international student body situated in the Middle East. The self-administered questionnaires: Clance's Impostor Phenomenon Scale (CIPS) and Rosenberg's Self-Esteem Scale (RSES) were completed by 290 medical students (58.3% females). Participants' median (range) age was 19 years (16–35). Students were of 28 different nationalities; the largest proportions were from Gulf Corporation Council (GCC) countries. The prevalence of low self-esteem was 18.6%, while 45.2% of the students demonstrated traits suggestive of IP. There was a strongly negative correlation between CIPS and RSES (*r* = −0.71). No significant gender differences were found in IP. Similarly, no differences in IP were found when comparing between age groups, previous experience in higher education or year of study. Multivariate analysis showed that students from GCC countries had higher levels of self-esteem relative to students from other regions. Low self-esteem was a strong predictor of IP. Country of origin may influence students' self-esteem studying in international university settings.

## Introduction

Students may perceive medical college as a place where skills, performance, and academic ability come under constant scrutiny. Competitive academic college environments position high-achieving students at risk of developing feelings of intellectual fraudulence and phoniness (1–3). The exhibition of these types of “impostor” characteristics has been associated with negative personality traits (4, 5), as well as anxiety and depression (6, 7). Impostor phenomenon (IP), as described by Clance & Imes in 1978, refers to the widely recognized fear of being exposed as a fraud (7), despite having validated self-achievement. Individuals with impostor feelings consider themselves as having deceived others into believing that they are more intelligent and more capable than they really are (8). They often attribute their accomplishments to luck, fate, personal charm, and attractiveness (9). In the workplace, IP has been associated with poor performance, job dissatisfaction, and burnout in several professions (10), including healthcare (6, 11).

Within student populations, self-esteem has been reported to be a principal precondition (12–14) and predictor of IP (15). Self-esteem being the evaluative dimension of self-knowledge, referring to how a person positively or negatively appraises themselves (16). Good mental health, competence, confidence and productivity all correlate positively with self-esteem (17, 18), whilst feelings of inferiority, sadness, depression, desperation and suicidal ideation are all associated with low self-esteem (19, 20). Individuals with high self-esteem demonstrate an acceptance of themselves as they are, regardless of their strengths and weaknesses. Therefore, they are less vulnerable to pressures to prove their worth (21). Neureiter and Traut-Mattausch investigated psychological barriers to a successful career-development process and suggested that imposter feelings are wrought by low self-esteem and both the fear of failure and the fear of success. The latter being more significant among a qualified workforce rather than among university students (12). This implies that low self-esteem may be predictive of IP at all stages of a skilled-worker's personal and professional development but it has a particular prominence at the undergraduate stage of education and training. Furthermore, a study of students categorized as impostors suffered more significant reductions in self-esteem than non-impostor students following subjective failure on a mid-term exam (15). In contrast, imposters did not differ from non-impostors after subjective success (15). These issues underline the importance of understanding the relationship between self-esteem and imposter feelings among all undergraduate students.

Academic achievement in medicine is attainable by individuals undeterred by IP (22, 23). However, it has been reported that 27.5% of healthcare students experienced IP (1), imposterism has been associated with burnout (8, 11, 24), and the feelings of self-doubt that accompany real and imagined underperformance (25). Insecurities about performance decrease with experience and career progression, but IP fosters feelings of incompetence at different stages across a doctor's career, including early stages of medical studies (24, 25). Admission to medical school is conditional upon students having high academic accomplishments, acting as concrete validation of aptitude. However, the nature and the sheer volume of the material comprising medical curricula may leave some students doubting their own academic abilities.

Experiences of racial discrimination have been associated with IP feelings by evoking a sense of “otherness” and thus reinforcing feelings of intellectual inferiority (5). Exposure to unfamiliar environments may exacerbate this sense of otherness, especially among women and underrepresented minorities (3, 7, 9, 11, 26, 27). The fear of being exposed as an “impostor” is a situational affective response, i.e., the presence or absence of IP is governed by the setting and the circumstance (28). A Canadian study found that foreign-trained residents were more susceptible to IP than graduates from medical colleges in Canada (11), implying that internationally trained doctors may have stronger feelings of IP than colleagues trained within their own country of citizenship. International medical colleges often have culturally diverse student populations. In these environments, individuals may find themselves emersed in a cultural milieu alien to their societies of origin (29), leading to further isolation and increased IP (3, 7, 9, 11, 26, 27).

There is disparity in the educational research literature regarding the strength of the relationship between IP and self-esteem measured in student populations (21, 30, 31). However, the various studies addressing this topic were not conducted among medical students, nor were they conducted in a university situated in the Middle East with a large Arab student body. To our knowledge, there are no published studies reporting upon IP and self-esteem among medical students in a predominantly Arabic speaking country.

We hypothesized that IP would be relatively high among medical students at a campus of an Irish medical college situated in the Middle East and that IP would be strongly predicted by low self-esteem. This study aimed at describing the prevalence of IP and low self-esteem within a medical college and quantifying the relationship between the two. Further, we assessed differences between subgroups of students regarding IP and self-esteem, focusing on gender, Arab/non-Arab, and domestic/international student status.

## Materials and Methods

### Study Design

This cross-sectional descriptive study was conducted in the Bahrain campus of the Royal College of Surgeons in Ireland. Participants were recruited from students enrolled on the 5-year medical program which consists of an integrated curriculum. The first and second years of study predominately focus on pre-clinical themes and are delivered within the college campus setting. From the third year of study until the completion of the course, educational themes are predominately clinical and are taught mainly in the clinical setting. The study included students from all 5-year groups. Recruitment took place before regularly timetabled teaching sessions in the school of medicine during the first semester of the academic year. Blank paper copies of the questionnaires were distributed to students at the beginning of their class and a verbal description of the research study was provided by one of the investigators. Students returned the completed questionnaire immediately after class.

### Participants

There were 770 students enrolled in the medical degree program spanning across 5-year groups (first to fifth year of study). There were approximately 154 students in each year group. Convenience sampling was used to approach students attending teaching activities that took place on the college campus. Students attending clinical teaching sessions outside of the campus were not approached. A total of 360 students were approached and received copies of the questionnaires. These were distributed at the beginning of a teaching activity. Of these 360 questionnaires, 295 were returned for a total response rate of 82%. Five questionnaires were handed back with incomplete responses giving a final number of analyzed questionnaires of 290. The median (range) age of participants was 19 years (1, 16–34). There were 119 males (41.0%), and 169 females (58.3%) spread over the first to fifth year of study groups. Six (2.1%) of the participants were married and 34 (11.7%) had obtained a degree before joining the medical program (Table 1). Students were of 28 different nationalities. The largest proportion of students were citizens of Bahrain, these were considered “domestic” students and all others were considered “international” students. The nationalities of the international students were grouped into three broad categories: Gulf Corporation Council (GCC) nationalities (i.e., Bahrain, Kuwait, Oman, Saudi Arabia and the United Arab Emirates), North American (Canada and USA) and others (Australia, Bangladesh, Egypt, France, Ghana, Iceland, Ireland, India, Italy, Jordan, Libya, Morocco, Pakistan, Palestine, South Africa, Spain, Syria, Iraq, UK, and Yemen).

**Table 1:** Participant and year of study group demographics.

	**Pre-Clinical**		**Clinical**		
**Year of study**	**First**	**Second**	**Third**	**Fourth**	**Fifth**
	***N* (%)**	***N* (%)**	***N* (%)**	***N* (%)**	***N* (%)**
**Gender**
Male	27 (56.2)	19 (30.1)	18 (56.2)	35 (41.2)	20 (33.3)
Female	21 (43.8)	44 (69.8)	14 (43.8)	50 (58.8)	40 (66.6)
**Not reported** **=** **2**
**Age**
<21 years old	43 (87.8)	46 (73.0)	18 (56.2)	31 (36.5)	7 (11.9)
>21 years old	6 (12.2)	17 (27.0)	14 (43.8)	54 (63.5)	52 (88.1)
**Not reported** **=** **2**
**Marital status**
Unmarried	47 (100)	62 (100)	31 (96.9)	79 (96.3)	58 (96.7)
Married	0 (0)	0 (0)	1 (3.1)	3 (3.7)	2 (3.3)
**Not reported** **=** **7**
**Previous education**
No degree	40 (85.1)	47 (74.6)	24 (8)	80 (97.6)	57 (95.0)
Degree	7 (14.9)	16 (25.4)	6 (2)	2 (2.4)	3 (5.0)
**Not reported** **=** **8**
**Domestic or international**
Domestic	18 (36.7)	30 (48.4)	9 (28.1)	48 (57.1)	25 (41.7)
International	31 (63.3)	32 (51.6)	23 (71.9)	36 (42.9)	35 (58.3)
**Not reported** **=** **3**
**Geographical region**
GCC	26 (53.1)	34 (54.8)	13 (40.6)	58 (69.0)	35 (58.3)
North America	6 (12.2)	12 (19.4)	10 (31.3)	7 (8.3)	9 (15.0)
Others	17 (34.7)	16 (25.8)	9 (28.1)	19 (22.6)	16 (26.7)
**Not reported** **=** **3**

No incentive for participation was provided. Participation was voluntary and written informed consent was collected from all participants before enrolment onto the study. The study was approved by Research Ethics Committee of RCSI Bahrain (reference: approval letter dated, October 22, 2019).

### Measures

Self-administered questionnaires of the Clance's Impostor Phenomenon Scale (CIPS) and Rosenberg Self-Esteem Scale (RSES) were used for this study. The two different tools were combined into one sheet and distributed together as a single questionnaire. The English version of both surveys was used.

Characteristics of the impostor phenomenon were assessed using CIPS (32). This survey consists of 20 items that each individual participant rates on a 5-point Likert scale (1; not at all true, 2; rarely true, 3; sometimes true, 4; often true, 5; very true). This results in a total score ranging from 20 to 100. The higher the score the more severe the manifestation of IP. A cut-off value of <63 was used to define an individual as an impostor (1, 3, 33). The CIPS has been reported to have a high internal reliability with Cronbach's α of 0.92 (33), and 0.96 (32). The current study gave a Cronbach's alpha coefficient of 0.87.

Self-esteem was assessed using RSES. This is a 10-item questionnaire, each having a 4- point Likert scale (0; strongly agree, 1; agree, 2; disagree, 3; strongly disagree). The total scoring ranges from 0 to 30 (34). The higher the score the more self-esteem the individual has. A cut-off value of <16 was used to define an individual as having low self-esteem. RSES has reported Cronbach alpha coefficients ranging from 0.87 to 0.92 for American college students (34). The current study gave a Cronbach's alpha coefficient of 0.86.

### Analysis

Returned completed questionnaires were coded with a specific sequential number. Data from each questionnaire returned was entered on to SPSS software package (IBM Corp. Released 2020. IBM SPSS Statistics for Windows, Version 27.0. Armonk, NY: IBM Corp). Descriptive statistics (mean, standard deviation, frequency, and percentage) were used to summarize data. Pearson Correlation was used to relate CIPS to RSES. Chi-squared test was used to risk of high CIPS as predicted by low RSES. Mean CIPS and mean RSES between variables were compared using *t*-test or ANOVA where appropriate. Median CIPS and RSES were compared for married and single groups using Mann-Whitney U test. Multivariate analysis (MANOVA) was used to predict demographic and personal variables affecting IP and self-esteem. The reliability of CIPS and RSES was assessed using Cronbach's alpha coefficient with a value of 0.70 or above considered as indicative of acceptable reliability. The statistical level of significance was set at *p* ≤ 0.05.

## Results

**Figure 1:**
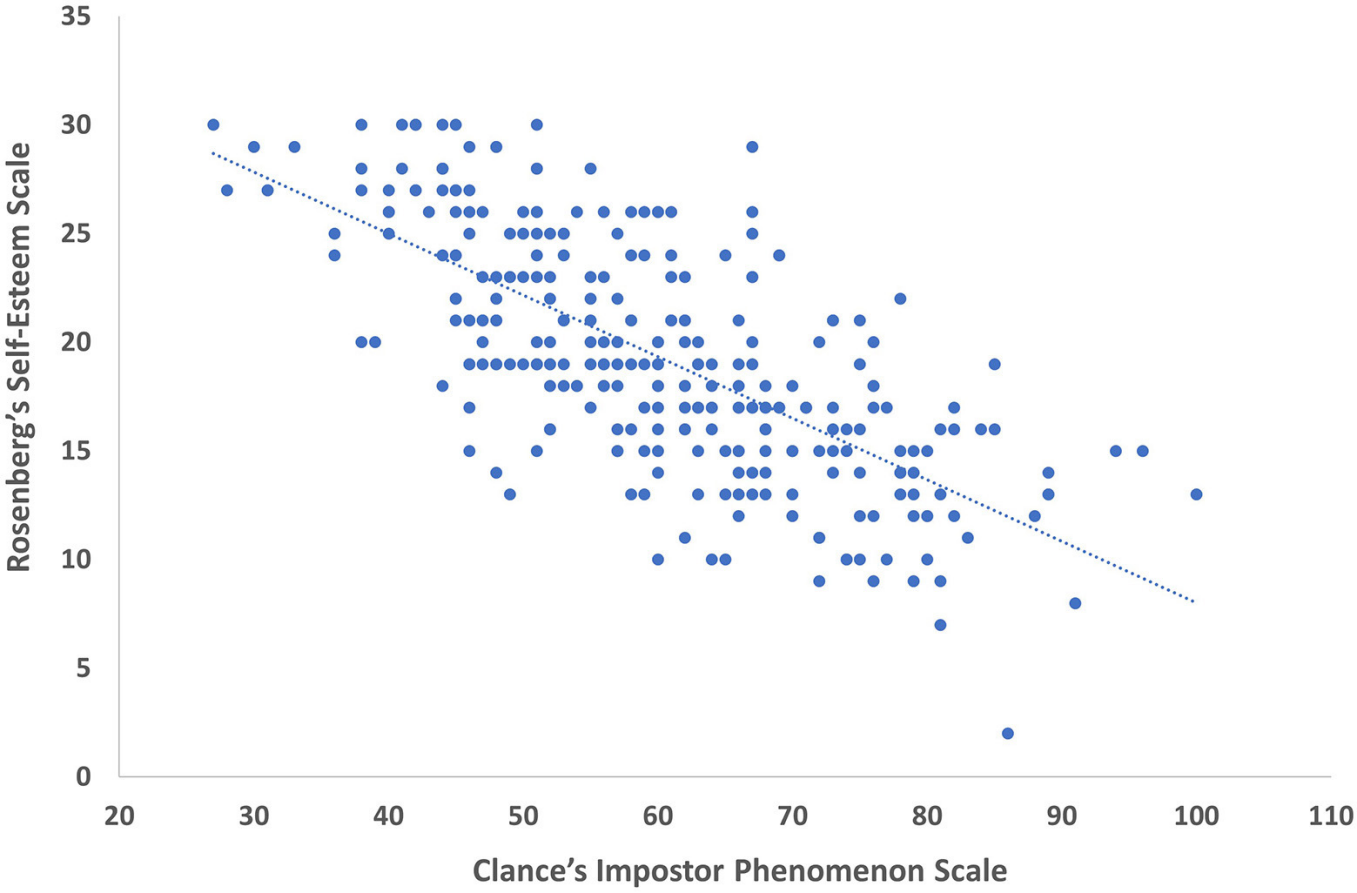
Correlation between student scores for clance impostor phenomenon scale and Rosenberg Self-Esteem Scale.

There were 131 (45.2%) participants who were imposters (i.e., high CIPS) and 54 (18.6%) participants classified as having low self-esteem (i.e., low RSES). The comparison of CIPS with RSES revealed a Pearson Correlation *r* = −0.71, *p* < 0.001 (Figure 1). There was a significant difference in the average CIPS between groups of low self-esteem (mean = 73.7 ± 10.3) and normal self-esteem (mean =57.0 ± 12.2) students (t_(288)_ = 9.3, *p* < 0.001). The risk of being an imposter was strongly associated with having low self-esteem. The frequencies of participants with low self-esteem and impostors gave an Odds Ratio of 1.5 (1.3–1.7), χ^2^_(1, N = 290)_ = 51, *p* < 0.001.

There were no significant differences in the average CIPS between demographic groups: males and females, <21 and >21 years of age, degree holders and non-degree holders, year of study groups, pre-clinical and clinical, domestic and international and geographical regions. Similarly, there were no significant differences in the average RSES between the listed demographic groups. These values are summarized in Table 2, Table 3. Also, there were no significant differences observed in the ratio of impostors between any of the demographic groups. There was a significant difference in the ratio of participants with low self-esteem between domestic students and foreign students, $\chi_{\left(1,\text{N}=287\right)}^{2}$ = 6.6, *p* < 0.01. However, there were no other such differences observed in frequencies of individuals with low self-esteem for the other listed demographics (see Table 2, Table 3). The study found small but significant differences in CIPS and RSES between married and unmarried students according to the Mann-Whitney *U*-test. Median (minimum–maximum) CIPS were: single 60 (27–100), and married 47 (42–66), *p* = 0.026. Median (minimum–maximum) RSES were: single 19 (2–30), married 24 (19–30), *p* = 0.040.

**Table 2:** Mean student's scores on the clance impostor phenomenon scale and frequency of imposterism.

		**Score**		**Imposter frequency**	**OR for imposter**
	***N* (%)**	**Mean (SD)**	***p*-value**	***N* (%)**	
Overall	290	60 (13.5)		131 (45.2)	
**Gender**
Male	119 (41.0)	59 (13.4)	0.129	46 (38.7)	1.57 (0.97–2.53)
Female	169 (58.3)	61 (13.5)		84 (49.7)	
Not reported	2 (0.7)				
Total	290 (100.0)				
**Age**
<21	145 (50.0)	60 (12.7)	0.766	63 (43.4)	1.18 (0.74–1.88)
>21	143 (49.3)	60 (14.3)		68 (47.6)	
Not reported	2 (0.7)				
Total	290 (100.0)				
**Marital status**
Unmarried	277 (95.5)	61 (13.4)	0.040	129 (46.6)	0.23 (0.03–1.99)
Married	6 (2.1)	49 (9.3)		1 (16.7)	
Not reported	7 (2.4)				
Total	290 (100.0)				
**Previous educations**
No degree	248 (85.5)	61 (13.4)	0.152	118 (47.6)	0.53 (0.25–1.13)
Degree	34 (11.7)	57 (14.0)		11 (32.4)	
Not reported	8 (2.8)				
Total	290 (100.0)				
**Year of study**
First	49 (16.9)	60 (12.8)	0.279	21 (42.9)	
Second	63 (21.7)	60 (12.4)		28 (44.4)	
Third	32 (11.0)	56 (12.8)		9 (28.1)	
Fourth	86 (29.7)	60 (13.8)		39 (45.3)	
Fifth	60 (20.7)	62 (15.0)		34 (56.7)	
Total	290 (100.0)				
**Place in the program**
Pre-clinical	144 (49.7)	59 (12.6)	0.154	58 (40.3)	1.48 (0.93–2.36)
Clinical	146 (50.3)	61 (14.3)		73 (50.0)	
Total	290 (100.0)				
**Domestic or international**
Domestic	130 (44.8)	59 (13.8)	0.139	51 (39.2)	1.57 (0.98–2.51)
International	157 (54.1)	61 (13.3)		79 (50.3)	
Not reported	3 (1.0)				
Total	290 (100.0)				
**Geographical region**
GCC	166 (57.2)	59 (14.1)	0.387	71 (42.8)	
North America	44 (15.2)	60 (13.2)		21 (47.7)	
Others	77 (26.6)	62 (12.4)		38 (49.4)	
Not reported	3 (1.0)				
Total	290 (100.0)				

*OR, odds ratio; SD, standard deviation*.

**Table 3:** Mean student's scores on the Rosenberg's Self-Esteem Scale and frequency of low self-esteem.

		**Score**		**Low self-esteem frequency**	**OR for low self-esteem**
	***N* (%)**	**Mean (SD)**	***p*-value**	***N* (%)**	
Overall	290	19 (5.4)		54 (18.6)	
**Gender**
Male	119 (41.0)	20 (5.6)	0.325	24 (20.2)	1.17 (0.64–2.13)
Female	169 (58.3)	19 (5.3)		30 (17.8)	
Not reported	2 (0.7)				
Total	290 (100.0)				
**Age**
<21	145 (50.0)	20 (5.0)	0.439	21 (14.5)	0.59 (0.32–1.08)
>21	143 (49.3)	19 (5.9)		32 (22.4)	
Not reported	2 (0.7)				
Total	290 (100.0)				
**Marital status**
Unmarried	277 (95.5)	19 (5.4)	0.042	54 (19.5)	1.03 (1.00–1.05)
Married	6 (2.1)	24 (4.6)		0 (0.0)	
Not reported	7 (2.4)				
Total	290 (100.0)				
**Previous educations**
No degree	248 (85.5)	19 (5.4)	0.396	48 (19.4)	1.39 (0.51–3.78)
Degree	34 (11.7)	20 (5.6)		5 (14.7)	
Not reported	8 (2.8)				
Total	290 (100.0)				
**Year of study**
First	49 (16.9)	19 (5.6)	0.788	11 (22.4)	
Second	63 (21.7)	20 (4.4)		6 (9.5)	
Third	32 (11.0)	20 (4.9)		5 (15.6)	
Fourth	86 (29.7)	19 (6.0)		19 (22.1)	
Fifth	60 (20.7)	19 (5.7)		13 (21.7)	
Total	290 (100.0)				
**Place in the program**
Pre-clinical	144 (49.7)	20 (4.9)	0.459	22 (15.3)	0.64 (0.35–1.17)
Clinical	146 (50.3)	19 (5.9)		32 (21.9)	
Total	290 (100.0)				
**Domestic or international**
Domestic	130 (44.8)	20 (5.1)	0.004	16 (12.3)	0.44 (0.23–0.83)
International	157 (54.1)	18 (5.9)		38 (24.2)	
Not reported	3 (1.0)				
Total	290 (100.0)				
**Geographical region**
GCC	166 (57.2)	20 (5.3)	0.010	25 (15.1)	
North America	44 (15.2)	19 (5.3)		8 (18.2)	
Others	77 (26.6)	18 (5.5)		21 (27.3)	
Not reported	3 (1.0)				
Total	290 (100.0)				

*OR, odds ratio; SD, standard deviation*.

One-way multivariate analysis of variance (MANOVA) was performed to assess one or more mean differences between various demographics (gender, marital status, previous degree, North American nationality, GCC nationality, pre-clinical, age below 21 years, year of course and domestic student status) and measures of IP and self-esteem. Preliminary tests were conducted to check for multicollinearity, multivariate normality, absence of multivariate outliers, homogeneity of variances; the results indicated that there were no serious violations of the MANOVA assumptions. Prior to conducting follow-up ANOVAs, the homogeneity of variance assumption was tested for CIPS and RSES. Based on Levene's *F*-tests, the homogeneity of variance assumption was considered satisfied (*p* > 0.05). Scheffe's *post hoc* test was used for multiple group comparisons. There was a significant difference between domestic students (Bahraini) and international students, Wilke's Λ =0.97, F_(2, 284)_ = 4.40,*p* = 0.01. There was a significant difference between domestic students and international students for RSES, F_(1, 285)_ = 8.3, *p* < 0.01), with domestic students (Mean = 20.3 ± 5.1) scoring higher than international students (mean = 18.5 ± 5.6). Further, there was a significant difference between geographical regions [GCC, North American and “others” (i.e. non-GCC and non-North American)] when considering jointly the variables CIPS and RSES. Wilke's Λ =0.96, F_(4, 566)_=2.68,*p* = 0.03. There were significant differences between GCC students, North American students and others for RSES, F_(2, 284)_ = 4.7, *p* = 0.01, with GCC students (mean = 20.0 ± 5.3) scoring higher than North American students (mean = 19.4 ± 5.3) and others (mean =17.7 ± 5.5). *Post-hoc* ANOVA revealed a significant difference between GCC student and others but no difference between GCC students and Northern American students for self-esteem scores only. No other significant differences were found using multivariate analysis.

## Discussion

This study explored the relationship between IP and self-esteem amongst medical students in a European medical college campus situated in the Arabic speaking Kingdom of Bahrain. CIPS were higher among students with low self-esteem. We found no significant gender differences in IP in this cohort. Similarly, we found no differences in IP between: age groups (<21 and >21 years of age), previous experience in higher education, year of study or domestic vs. international student status. Domestic-status and citizenship of a GCC country was associated with self-esteem.

IP was first described in highly performing women (7). Subsequent studies have shown IP to affect both genders equally in the professional setting (8). However, studies among medical students have demonstrated female gender to be associated with IP (1, 24). The present study found no such associations with gender. Major professional or educational transitions are crucial periods when IP is likely to occur (2, 25), particularly in the third-year (classroom to clinic) transition (1, 2, 24). The present study found no significant differences in CIPS between the years of study, including the critical third-year. We found a small but statistically significant difference in CIPS between married and unmarried students. However, the number of married students (*n* = 6) was relatively low. Henning et al., had shown that married medical students had less distress than their single counterparts (1), and it has been suggested that marriage might act as a buffer against distress (35).

Multiple factors may interplay to explain the relationship between self-esteem and IP. The development of impostor traits may stem from innate predispositions (7, 36), familial and developmental. These include personality traits (e.g., neurotic personality traits, perfectionism etc.,) that are linked to perpetuating feelings of low self-esteem (37).

Students from GCC countries and North America had relatively high self-esteem. These two categories are well defined geopolitically. In contrast, our category of “other” (i.e., non-American, -Canadian or -GCC nationalities) was geographically and socially very diverse. The interrelationships between culture, nationality, self-esteem and personality have previously been commented upon. Fulmer et al. (29), explored the concept of “person-culture match” which predicts that “when a person's personality matches the prevalent personalities of other people in a culture, culture functions as an amplifier of the positive effect of personality on self-esteem” (29). Students who are raised in GCC countries share many cultural features and ideals and have shared historical backgrounds. Abdulkhaliq et al. (16), studied self-esteem amongst college students in four different Arab countries and reported self-esteem to be higher in students from countries with high per-capita incomes. This reflects our findings of high self-esteem among students from economically advanced countries. Further, issues of race and ethnicity may also be important in understanding the differences in the levels of self-esteem and impostor traits. Bernard et al. studied the effect of racial identity on impostor traits in minorities and concluded that a high sense of racial identity generally confers protection against developing impostor traits (5). Although, our study did not examine ethnicity as a subgroup, it is postulated that non-GCC students may experience feelings related to being a racial minority while studying in Bahrain. This may contribute toward levels of self-esteem.

Our findings highlight the importance of identifying individuals with IP in order to optimize their learning experiences and professional progression. Clance and Imes recommended frequent, specific feedback as a solution for those who doubt their abilities (38). Future investigations may focus on relating IP to “educational safety” and learning in non-judgmental settings. Eliminating unhealthy competitiveness in campus culture and creating environments where students are supported educationally and personally may reduce IP (39). International medical colleges should be attentive to the needs of minorities in order to reduce IP and allow diversity to be a universal asset to the educational experience (40, 41).

Limitations of this study include our use of convenience sampling of medical students who may not represent the full student body. The timing of the study overlapped with the examination preparation period, and so attendance of the classes was lower than expected. Minimal demographic data were collected for non-respondents preventing us from assessing the degree to which selection bias might have occurred. As this is a cross-sectional study, no causal conclusions can be made. The subgroups of married students and post-graduate students had relatively low numbers of participants.

Low self-esteem was found to be a predictor of IP with medical students. Female students were no more likely than males to display characteristics of IP. Previous university experience, year of study on the medical program and age did not significantly affect measures of IP. Domestic student status and citizenship of a GCC or a North American country affected self-esteem. Foreign/international students may benefit from targeted support to address students' self-esteem and its negative consequences on professional performance and personal wellbeing. International medical colleges should implement general support measures for domestic and international students. These measures should be aimed at making the general culture of the college more acceptable to students from diverse backgrounds, particularly for non-citizens of the country in which the campus is located.

## Data Availability Statement

The raw data supporting the conclusions of this article will be made available by the authors, without undue reservation.

## Ethics Statement

The studies involving human participants were reviewed and approved by Research Ethics Committee of RCSI Bahrain. The patients/participants provided their written informed consent to participate in this study.

## Author Contributions

MN and SF: conceptualization and visualization. SF: data curation, formal analysis, and validation. MN, NH, MZ, AZ, and YM: investigation. MN, NH, and MZ: methodology, project administration, resources, and supervision. MN, NH, MZ, and SF: writing–original manuscript. MN, NH, MZ, AZ, YM, and SF: writing–review and editing. All authors contributed to the manuscript and approved the submitted version.

## Conflict of Interest

The authors declare that the research was conducted in the absence of any commercial or financial relationships that could be construed as a potential conflict of interest.

## Publisher's Note

All claims expressed in this article are solely those of the authors and do not necessarily represent those of their affiliated organizations, or those of the publisher, the editors and the reviewers. Any product that may be evaluated in this article, or claim that may be made by its manufacturer, is not guaranteed or endorsed by the publisher.

## Abbreviations

ANOVA: Analysis of variance
CIPS: Clance's Impostor Phenomenon Scale
GCC: Gulf Corporation Council countries
IP: Imposter phenomenon
MANOVA: Multivariate analysis of variance
RSES: Rosenberg's Self-Esteem Scale.

## References

[1] Henning K Ey S Shaw D. Perfectionism, the imposter phenomenon and psychological adjustment in medical, dental, nursing and pharmacy students. Med Educ. (1998) 32:456–64. 10.1046/j.1365-2923.1998.00234.x 10211285

[2] Levant B Villwock JA Manzardo AM. Impostorism in third-year medical students: an item analysis using the Clance impostor phenomenon scale. Perspect Med Educ. (2020) 9:83–91. 10.1007/s40037-020-00562-8 32030630 PMC7138782

[3] September AN McCarrey M Baranowsky A Parent C Schindler D. The relation between well-being, impostor feelings, and gender role orientation among Canadian university students. J Soc Psychol. (2001) 141:218–32. 10.1080/00224540109600548 11372567

[4] Bernard NS Dollinger SJ Ramaniah NV. Applying the big five personality factors to the impostor phenomenon. J Pers Assess. (2002) 78:321–33. 10.1207/S15327752JPA7802_07 12067196

[5] Bernard DL Hoggard LS Neblett EW. Racial discrimination, racial identity, and impostor phenomenon: a profile approach. Cultur Divers Ethnic Minor Psychol. (2018) 24:51–61. 10.1037/cdp0000161 28414495

[6] Oriel K Plane MB Mundt M. Family medicine residents and the impostor phenomenon. Fam Med. (2004) 36:248–52. 15057614

[7] Clance PR Imes SA. The imposter phenomenon in high achieving women: Dynamics and therapeutic intervention. Psychol Psychother Theory Res Pract. (1978) 15:241. 10.1037/h0086006

[8] Leach PK Nygaard RM Chipman JG Brunsvold ME Marek AP. Impostor phenomenon and burnout in general surgeons and general surgery residents. J Surg Educ. (2019) 76:99–106. 10.1016/j.jsurg.2018.06.025 30122638

[9] Clance PR. O'toole MA. The imposter phenomenon: An internal barrier to empowerment and achievement. Women Therapy. (1987) 6:51–64. 10.1300/J015V06N03_05

[10] Bravata DM Watts SA Keefer AL Madhusudhan DK Taylor KT Clark DM. Prevalence, Predictors, and Treatment of Impostor Syndrome: a Systematic Review. J Gen Intern Med. (2020) 35:1252–75. 10.1007/s11606-019-05364-1 31848865 PMC7174434

[11] Legassie J Zibrowski EM Goldszmidt MA. Measuring resident well-being: impostorism and burnout syndrome in residency. J Gen Intern Med. (2008) 23:1090–4. 10.1007/s11606-008-0536-x 18612750 PMC2517942

[12] Neureiter M Traut-Mattausch E. An Inner Barrier to Career Development: Preconditions of the Impostor Phenomenon and Consequences for Career Development. Front Psychol. (2016) 7:48. 10.3389/fpsyg.2016.00048 26869957 PMC4740363

[13] Jöstl G Bergsmann E Lüftenegger M Schober B Spiel C. When will they blow my cover? The impostor phenomenon among Austrian doctoral students. Zeitschrift für Psychologie. (2012) 220:109–20. 10.1027/2151-2604/a000102

[14] Vergauwe J Wille B Feys M De Fruyt F Anseel F. Fear of being exposed: the trait-relatedness of the impostor phenomenon and its relevance in the work context. J Bus Psychol. (2015) 30:565–81. 10.1007/s10869-014-9382-5

[15] Cozzarelli C Major B. Exploring the validity of the impostor phenomenon. J Soc Clin Psychol. (1990) 9:401–17. 10.1521/jscp.1990.9.4.401

[16] Abdel-Khalek AM Korayem AS El-Nayal MA. Self-esteem among college students from four Arab countries. Psychol Rep. (2012) 110:297–303. 10.2466/07.09.17.PR0.110.1.297-303 22489395

[17] Rosenberg M. Society and the Adolescent Self-Image. Princeton, NJ: Princeton University Press (2015).

[18] Maslow AH. Motivation and Personality (1987).

[19] Taylor SE Brown JD. Illusion and well-being: a social psychological perspective on mental health. Psychol Bull. New York, NY: Harper Collins (1988) 103:193. 10.1037/0033-2909.103.2.193 3283814

[20] Battle J. Relationship between self-esteem and depression among high school students. Percept Mot Skills. (1980) 51:157–8. 10.2466/pms.1980.51.1.157 7432952

[21] Schubert N Bowker A. Examining the impostor phenomenon in relation to self-esteem level and self-esteem instability. Current Psychology. (2019) 38:749–55. 10.1007/s12144-017-9650-4

[22] Shreffler J Weingartner L Huecker M Shaw MA Ziegler C Simms T. Association between characteristics of impostor phenomenon in medical students and step 1 performance. Teach Learn Med. (2020) 33:36–48. 10.1080/10401334.2020.1784741 32634054

[23] Levant B Villwock JA Manzardo AM. Impostorism in American medical students during early clinical training: gender differences and intercorrelating factors. Int J Med Edu. (2020) 11:90–6. 10.5116/ijme.5e99.7aa2 32356519 PMC7246127

[24] Villwock JA Sobin LB Koester LA Harris TM. Impostor syndrome and burnout among American medical students: a pilot study. Int J Med Edu. (2016) 7:364–9. 10.5116/ijme.5801.eac4 27802178 PMC5116369

[25] LaDonna KA Ginsburg S Watling C. “Rising to the level of your incompetence”: what physicians' self-assessment of their performance reveals about the imposter syndrome in medicine. Acad Med. (2018) 93:763–8. 10.1097/ACM.0000000000002046 29116983

[26] Chrousos GP Mentis AA. Imposter syndrome threatens diversity. Science. (2020) 367:749–50. 10.1126/science.aba8039 32054753

[27] Sturges D. Imposter phenomenon and underrepresented minorities: what physician assistant educators need to know. J Physician Assist Educ. (2018) 29:126–8. 10.1097/JPA.0000000000000194 29683917

[28] McElwee RO Yurak TJ. The phenomenology of the impostor phenomenon. Individ Differ Res. (2010) 8:184–97.

[29] Fulmer CA Gelfand MJ Kruglanski AW Kim-Prieto C Diener E Pierro A. On “feeling right” in cultural contexts: how person-culture match affects self-esteem and subjective well-being. Psychol Sci. (2010) 21:1563–9. 10.1177/0956797610384742 20876880

[30] Sonnak C Towell T. The impostor phenomenon in British university students: Relationships between self-esteem, mental health, parental rearing style and socioeconomic status. Pers Individ Dif. (2001) 31:863–74. 10.1016/S0191-8869(00)00184-7

[31] Cusack CE Hughes JL Nuhu N. Connecting gender and mental health to imposter phenomenon feelings. Psi Chi J Psychol Res. (2013) 18:74–81. 10.24839/2164-8204.JN18.2.74

[32] Chrisman SM Pieper WA Clance PR Holland CL Glickauf-Hughes C. Validation of the clance imposter phenomenon scale. J Pers Assess. (1995) 65:456–67. 10.1207/s15327752jpa6503_6 16367709

[33] Holmes SW Kertay L Adamson LB Holland CL Clance PR. Measuring the impostor phenomenon: a comparison of Clance's IP Scale and Harvey's I-P Scale. J Pers Assess. (1993) 60:48–59. 10.1207/s15327752jpa6001_3 8433268

[34] Sinclair SJ Blais MA Gansler DA Sandberg E Bistis K LoCicero A. Psychometric properties of the Rosenberg Self-Esteem Scale: Overall and across demographic groups living within the United States. Eval Health Prof. (2010) 33:56–80. 10.1177/0163278709356187 20164106

[35] Coombs RH Fawzy FI. The effect of marital status on stress in medical school. Am J Psychiatry. (1982) 139:1490–3. 10.1176/ajp.139.11.1490 7137402

[36] Katz C. If I'm So Successful, Why Do I Feel Like a Fake?: The Impostor Phenomenon. New York, NY: Pocket Books; Markham, Ont. Distributed in Canada by PaperJacks (1986).

[37] Robins RW Tracy JL Trzesniewski K Potter J Gosling SD. Personality correlates of self-esteem. J Res Pers. (2001) 35:463–82. 10.1006/jrpe.2001.2324

[38] Clance PR. The Impostor Phenomenon: Overcoming the Fear That Haunts Your Success. Atlanta, GA:Peachtree Pub Ltd. (1985).

[39] Tsuei SH Lee D Ho C Regehr G Nimmon L. Exploring the construct of psychological safety in medical education. Acad Med. (2019) 94:S28–35. 10.1097/ACM.0000000000002897 31365407

[40] Nivet MA. A Diversity 3.0 update: are we moving the needle enough? Acad Med. (2015) 90:1591–3. 10.1097/ACM.0000000000000950 26422594

[41] Ackerman-Barger K Boatright D Gonzalez-Colaso R Orozco R Latimore D. Seeking inclusion excellence: understanding racial microaggressions as experienced by underrepresented medical and nursing students. Acad Med. (2020) 95:758–63. 10.1097/ACM.0000000000003077 31725462 PMC7185051

