# Supplementary material for: Impostor Phenomenon and Its Relationship to Self-Esteem Among Students at an International Medical College in the Middle East: A Cross Sectional Study
Source: Front Med (Lausanne). 2022 Apr 4;9:850434. doi: 10.3389/fmed.2022.850434 (PMC9013881; doi:10.3389/fmed.2022.850434)
Supplement: Supplementary file 1 [file Table_1.DOCX]

Supplementary Table 1: Enrolled students (n=770) in the medical degree program spanning across 5-year groups (first to fifth year of study). Participant and year of study group demographics.

|  |  | | | Pre-Clinical | |  |  |  | | Clinical | | | |  |
| --- | --- | --- | --- | --- | --- | --- | --- | --- | --- | --- | --- | --- | --- | --- |
| Year of study | First | | |  | Second |  | Third | |  | | Fourth |  | Fifth |  |
|  | N | | | (%) | N | (%) | N | | (%) | | N | (%) | N | (%) |
| Total | 152 | | |  | 156 |  | 172 | |  | | 142 |  | 148 |  |
| Gender |  | | |  |  |  |  | |  | |  |  |  |  |
| Male | 72 | | | 47.1 | 71 | 45.3 | 96 | | 55.6 | | 65 | 45.9 | 79 | 53.2 |
| Female | 80 | | | 52.9 | 85 | 54.7 | 76 | | 44.4 | | 77 | 54.1 | 69 | 46.8 |
| Domestic or international | | |  |  |  |  |  | |  | |  |  |  |  |
| Domestic | 62 | | | 40.5 | 65 | 41.5 | 72 | | 42.0 | | 60 | 42.4 | 58 | 39.0 |
| International | 90 | | | 59.5 | 91 | 58.5 | 100 | | 58.0 | | 82 | 57.6 | 90 | 61.0 |
| Geographical region | |  | |  |  |  |  | |  | |  |  |  |  |
| GCC | 76 | | | 50.3 | 80 | 51.6 | 102 | | 59.3 | | 87 | 61.0 | 81 | 54.5 |
| North America | 27 | | | 17.6 | 29 | 18.9 | 31 | | 18.0 | | 17 | 11.9 | 33 | 22.1 |
| Others | 49 | | | 32.0 | 46 | 29.6 | 39 | | 22.7 | | 39 | 27.1 | 35 | 23.4 |
